# Catalase Detection via Membrane-Based Pressure Sensors

**DOI:** 10.3390/molecules29071506

**Published:** 2024-03-28

**Authors:** Monica Bianco, Alessandra Zizzari, Elisabetta Perrone, Diego Mangiullo, Marco Mazzeo, Ilenia Viola, Valentina Arima

**Affiliations:** 1 CNR NANOTEC—Institute of Nanotechnology, c/o Campus Ecotekne, Via Monteroni, 73100 Lecce, Italy; monica.bianco@nanotec.cnr.it (M.B.); alessandra.zizzari@nanotec.cnr.it (A.Z.); elisabetta.perrone@nanotec.cnr.it (E.P.); diego.mangiullo@nanotec.cnr.it (D.M.); 2 Department of Mathematics and Physics “E. De Giorgi”, University of Salento, 73100 Lecce, Italy; marco.mazzeo@unisalento.it; 3 CNR NANOTEC—Institute of Nanotechnology, S.Li.M Lab, c/o Department of Physics, Sapienza University, P.le A. Moro 5, 00185 Rome, Italy

**Keywords:** pressure sensors, PDMS membranes, catalase, bioassays, membrane deflection

## Abstract

Membrane-based sensors (MePSs) exhibit remarkable precision and sensitivity in detecting pressure changes. MePSs are commonly used to monitor catalytic reactions in solution, generating gas products crucial for signal amplification in bioassays. They also allow for catalyst quantification by indirectly measuring the pressure generated by the gaseous products. This is particularly interesting for detecting enzymes in biofluids associated with disease onset. To enhance the performance of a MePS, various structural factors influence membrane flexibility and response time, ultimately dictating the device’s pressure sensitivity. In this study, we fabricated MePSs using polydimethylsiloxane (PDMS) and investigated how structural modifications affect the Young’s modulus (*E*) and residual stress (*σ*_0_) of the membranes. These modifications have a direct impact on the sensors’ sensitivity to pressure variations, observed as a function of the volume of the chamber (*Σ*) or of the mechanical properties of the membrane itself (*S*). MePSs exhibiting the highest sensitivities were then employed to detect catalyst quantities inducing the dismutation of hydrogen peroxide, producing dioxygen as a gaseous product. As a result, a catalase enzyme was successfully detected using these optimized MePSs, achieving a remarkable sensitivity of (22.7 ± 1.2) µm/nM and a limit of detection (LoD) of 396 pM.

## 1. Introduction

Pressure sensors are important tools for achieving a high control over the progression of chemical reactions and overflow rates in “Lab-on-a-Chip (LoC)” platforms. Given the reduced space available for their integration, conventional mechanical pressure gauges are often located outside the LoCs, leading to complications in the experimental set-up. Although commercial systems are convenient, they are not appropriate for LoC miniaturized systems due to their limited response time and high-pressure range [1].

To overcome these problems, innovative miniaturized technologies for detecting pressure changes in LoCs are currently under study [2]. As common approach, the pressure-induced deformation of a membrane integrated to a LoC is detected using a variety of electrical, piezoresistive and optical methods [3,4,5,6,7,8,9,10].

The thickness of the membrane is usually in the range of hundreds or tens of micrometers, and the material is often polydimethylsiloxane (PDMS) because of its mechanical properties, optical transparency and easy manufacturing, which explain its widespread use in LoCs and microfluidics [11,12,13,14,15,16]. The pressure range of many integrated-membrane pressure sensors is of a few kPa [7,8,17].

In a previous work [18], we have monitored the dioxygen (O_2_) evolution resulting from catalytic hydrogen peroxide (H_2_O_2_) dismutation by using a membrane-based pressure sensor (MePS) operating in a range between 2 and 50 Pa. A MePS chip consists of an array of microchambers of 8 mm diameter, integrating a 2 µm thin PDMS membrane whose deflection as a response to pressure changes is detected using a high-resolution camera. The interest in H_2_O_2_ dismutation catalyzed by ruthenium-embedding microcapsules was due to its applications in pumping-free LoCs [19] and fast-mixing microfluidic platforms [20]. However, H_2_O_2_ dismutation induced by catalase-like catalysts is also widely exploited in the biosensing field to monitor glucose-driven cellular respiration [21] and to amplify the immunoassay signal in highly sensitive chips [22,23].

Among H_2_O_2_-fed biosensors, volumetric bar-chart chips (V-chips) have recently attracted attention as cheap and powerless microsystems for detecting circulating tumor cells [24,25] and myocardial infarction biomarkers in serum [25], aflatoxin B1 [26] and Ochratoxin A [27] in beer, and lead ions in biological and environmental samples [28]. In V-chips, O_2_ generation due to the dismutation of H_2_O_2_ is directly proportional to the amount of analytes that interact with a suitable probe conjugated with the catalyst [24,25,29,30] or that react with a gel (formed by the capture probe and the catalyst) by releasing a proportional amount of catalyst in the supernatant [22,26,27,28]. The gas production leads to a volumetric expansion that is readable through an integrated bar-chart.

On the other hand, H_2_O_2_-fed biosensors enable the direct quantification of enzymes producing gaseous products, like catalase, with an important role in biological defense systems [31]. In humans, abnormal levels of catalase are related to diseases such as diabetes [32], cancer [33], cardiovascular disease [34] and Alzheimer’s disease [35]. Among current studies of catalase detection, based on optical [36], amperometric [37] and colorimetric methods [38], liquid crystal-based sensing platforms have been proposed as cheap and sensitive tools for catalase sensing in human serum [39,40].

Here, we have developed a catalase sensor as a strategic component for LoC platforms, biosensors or biological assays. The sensor is composed of a MePS chip, optimized in its operation through the modulation of structural parameters such as the Young’s modulus (*E*) and residual stress (*σ*_0_) of the membrane it comprises. These parameters are crucial for determining the sensor’s sensitivity to pressure variations, both as a function of the chamber volume and the mechanical characteristics of the membrane itself (*Σ* and *S*, respectively). The use of MePS can, therefore, be finely directed toward the detection of small quantities of an enzyme that produces O_2_ during the dismutation of H_2_O_2_. The achieved results are explicative to develop catalase biosensors for diagnostic applications and immunological assays based on MePSs.

## 2. Results and Discussion

The design of the MePS has been optimized to enable the highly sensitive detection of catalase activity. To this aim, several types of MePSs differing in their chamber diameter, membrane thickness and internal volume have been produced and characterized (see Table 1 for a detailed description).

For each type of MePS, parameters like Young’s modulus *E*, residual stress *σ*_0_ and the sensitivities both of the chamber dimension and of the membrane (*Σ* and *S*, respectively) have been calculated (see Table 2).

The operational performance of MePS was evaluated in relation to its sensitivity to the most significant structural parameters: Young’s modulus *E* and residual stress *σ*_0_. *E* measures the elastic resistance of the membrane to deformation, influencing the sensitivity of the sensor and its response time. The residual stress *σ*_0_, which remains on the membrane after the initial stimulus has been removed, can influence the response of the MePS to long reaction times. This happens especially in the analysis of slow reactions and affects the re-usability of the chip itself. More importantly, the effect of residual stresses on the structural elements of the MePS-sensing device (i.e., membrane thickness or chamber volumes) could cause a loss of linearity and a deterioration of load capacity.

The sensitivity of the chamber dimension *Σ* = Δ*P*/Δ*V* represents the ratio between the variation of pressure applied and the increment in the loaded liquid volume. A high value of *Σ* indicates that, with the same volume loaded, the device can detect a greater variation in the reaction pressure as an output. The membrane sensitivity *S* = Δ*w*/Δ*P* of a MePS can be described as the minimum input of the internal pressure change that creates a detectable output of membrane deflection. MePS devices with higher *S* values are more efficient since they allow you to detect greater variations of membrane deflections with the analogous reaction pressures.

The reliability of a membrane sensor is ensured through two key factors: (i) maintaining a linear regime for deformations and residual stresses and (ii) exhibiting an elastic response to varying degrees of deformation. To identify the most efficient MePS from those listed in Table 1, this study involved the analysis of the structural parameters and characteristic curves obtained from bulge tests.

### 2.1. Bulge Tests Theory

**Figure 1:**
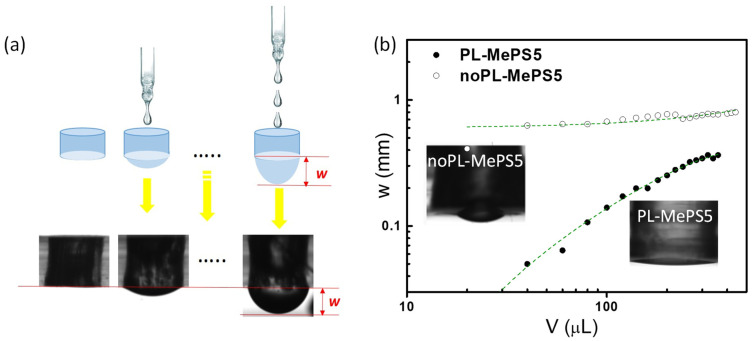
(**a**) Scheme of a MePS chip and of the method used to detect membrane deflection during the calibration steps of the bulge test (upper part). In the lower part, real images of the chip acquired by a high-resolution camera are shown; (**b**) graph in the log-log scale showing deflection (w) versus the water loaded volume (µL) during the bulge tests performed on two different devices with the same chamber dimensions (diameter of 8 mm) and membrane thickness (10 µm) but assembled without (noPL-MePS5, empty dots) or with the use of plasma treatment (PL-MePS5, full dots). The reported dotted lines better show the linear trend of PL-MePS. Images of membrane deflection of PL-MePS and no-PLMePS are reported as insets.

**Figure 2:**
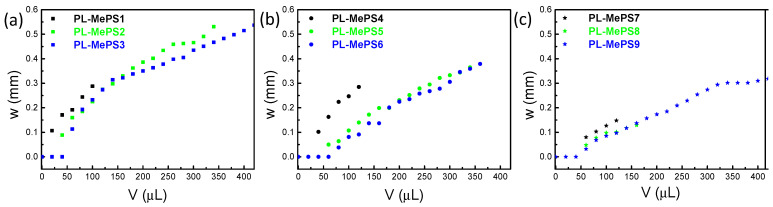
Graphs showing three different sets of measurements (deflection (w) versus the water volume (V) loaded in the chamber) acquired on PL-MePSs with variable chamber diameters: 5 mm (PL-MePS1; PL-MePS4; PL-MePS7); 8 mm (PL-MePS2; PL-MePS5; PL-MePS8); 10 mm (PL-MePS3; PL-MePS6; PL-MePS9); and membrane thicknesses fixed at (**a**) 2 µm, (**b**) 10 µm and (**c**) 50 µm.

The bulge test is a method used to evaluate the mechanical properties of a circular membrane by applying a uniform strain in the radial direction (Figure 1a). The membranes, assembled to the MePS chambers, are deformed by the weight force of water droplets added with aliquots of 10 or 20 μL volumes. The applied pressure *P* determines a hemispherical deflection of the membrane whose maximum w is measured by the data processing of an image acquired by a high-resolution camera. Typical plots of deflection *w* (mm) versus the added volume V (µL) are shown in Figure 1b and Figure 2. Considering that *P* in this specific situation is essentially due to the weight of the liquid volume, it can be calculated by applying the fundamental law of the hydrostatic pressure of a liquid by the action of gravity and the forces acting on the liquid surface (Stevino’s law), Equation (1):

(1) $$P=\rho \;g\;\left(h+w\right)$$

with *ρ* representing the water density, *g* the gravity acceleration and *h* the height of the water level in the chamber. The loading pressure *P* and the maximum membrane deflection *w* are related by the following relation, Equation (2):

(2) $$P=\frac{C_{1}d}{r^{2}}\;\sigma_{0}w+\frac{C_{2}f\left(\nu \right)d}{r^{4}}\;\frac{E}{1-\nu }w^{3}=Aw+Bw^{3}$$

where *r* is the membrane radius, *d* is its thickness, and *ν* is Poisson’s ratio. The geometrical coefficients *C*_1_, *C*_2_ and *f*(*v*) for circular membranes are 4, 2.67 and 1, respectively.

**Figure 3:**
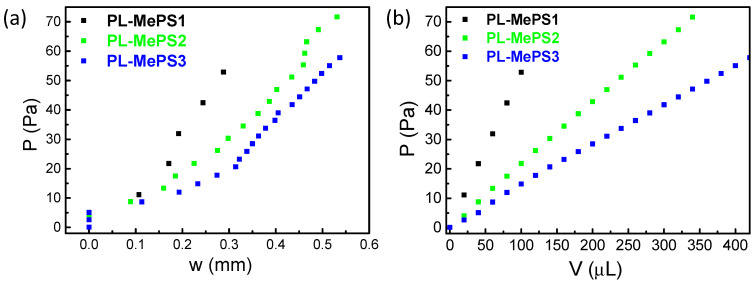
Plots from curves of Figure 2a to calculate (**a**) the residual stress *σ*_0_ and Young’s modulus *E*, (**b**) sensitivity of chamber dimension for PL-MePS1-3 with membrane thickness of 2 µm and diameter of 5, 8 and 10 mm, respectively.

Equation (2), written in a synthetic form with coefficients A and B, has been used as the fitting function of the *P*/*w* curves shown in Figure 3a, Figures S1a, S2a and S4b,c. A and B describe the elastic response of the membrane as the residual stress *σ*_0_ and Young’s modulus *E*.

**Figure 4:**
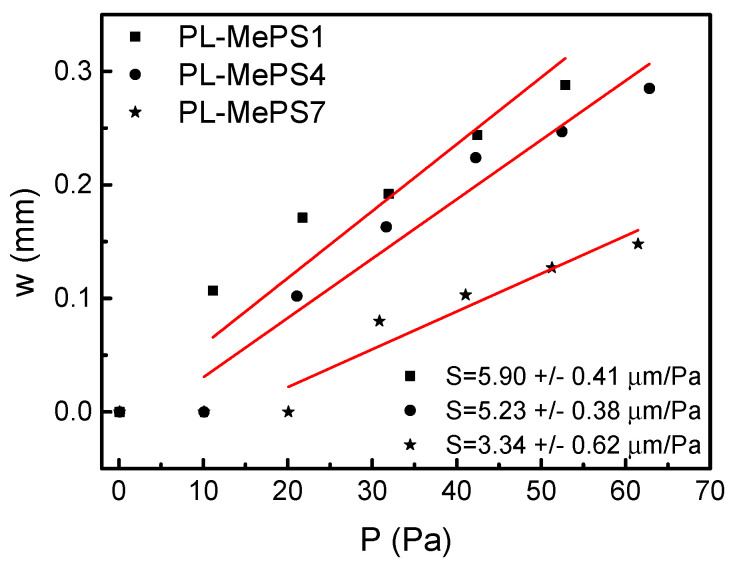
Plots from black curves of Figure 2 to calculate the sensitivity of the membrane *S* for PL-MePS1, PL-MePS4 and PL-MePS7 with membrane thicknesses of 2, 10 and 50 µm, respectively. The inset at the bottom reports the sensitivity values obtained by a fitting procedure.

On the other hand, by plotting the pressure *P* (Equation (1)) versus the water-loaded volume *V* (Figure 3b, Figure S1b and S2b) and the deflection *w* versus the pressure *P* (shown in Figure 4), the sensitivity of the chamber *Σ* (Pa/µL) and the sensitivity of the membrane *S* (µm/Pa) can be, respectively, calculated.

### 2.2. Plasma-Induced Effects on Membranes

In the integration process, two distinct methods were employed to incorporate the sensing membrane into the MePS chips: oxygen plasma treatment (PL-MePS) and a mortar layer (noPL-MePS) (details in Section 3.2 of Materials and Methods and par. “Plasma-induced effects on membranes” in Supplementary Materials). We found that the plasma treatment increased membrane hydrophilicity by modifying surface composition with the creation of a thin silica-like layer at the interface [41,42,43]. This enhanced hydrophilicity in PL-MePS chips led to more consistent curves with a broader linear range of responsivity. Therefore, subsequent studies were conducted exclusively using PL-MePS sensors. NoPL membranes, on the contrary, show reduced sensitivity due to non-linear behavior.

The reproducibility in the PL-MePSs is reported in Figure S4 and discussed in Supplementary Materials (par. “Curve reproducibility”). At lower pressures, some differences due to the fabrication process may cause a higher inertia to mechanical deformation [44]. A proper calibration of each MePS before use may eliminate this problem.

### 2.3. MePS Calibrations

Hence, all the plasma-treated MePSs produced have been tested. Their curves are reported in Figure 2. Thinner membranes deflect more efficiently than thicker ones at similar loading volumes. The behavior of the curves in the 8 and 10 mm diameter chambers sometimes overlaps. Membranes interfaced with smaller chambers deflect more than those interfaced with large-diameter chambers; indeed, for membranes with the same thickness, a fixed water volume exerts a larger pressure on a smaller surface (as in the 5 mm diameter chambers), thus determining larger deflections. Furthermore, membranes in larger chambers need higher volumes of liquid to overcome the original inertia to deflection (V > 50 µL) and show different slopes of the *w* trend as a function of loaded volume.

From the curves of Figure 2, further fittings have allowed us to evaluate the parameters above mentioned: Young’s modulus *E*, residual stress *σ*_0_ and the sensitivity of the chamber dimension *Σ*. The plots from which all these values have been calculated are reported in Figure 3 for PL-MePS1-3 with a membrane thickness of 2 µm and for all the other chips with thicker membranes (PL-MePS4-6 with 10 µm thick membrane and PL-MePS7-9 with 50 µm thick membrane) in Figures S1 and S2.

The results of all the fits are shown in Table 2. An average value of (1.75 ± 0.90) MPa is found for PL-MePS1, which is in agreement with previous works on plasma-treated PDMS membranes of similar thicknesses [45,46,47,48].

Considering *E*, a decrease in its absolute value is observed by increasing the thickness of the membrane. An explanation of the experimental differences in the *E* values can be attributed to the fabrication method of membranes. Indeed, PDMS membranes are well known to have Young’s modulus values larger than bulk PDMS [45,46,47,48] in a range between 12 kPa and 2.50 MPa, depending on the processing conditions. The differences have been attributed to the pre-stretching of the PDMS chains of the membranes produced by spin coating (at 6000 RPM for a relatively long time of up to 150 s) [44] compared to the relaxed state of the PDMS chains of membranes prepared by pouring on a flat substrate. Here, we believe that the higher rigidity of the 2 µm thin membranes of PL-MePS1 is due to the higher rotation speeds that are applied during the spin-coating fabrication procedure compared to 10 µm (PL-MePS2) and 50 µm thick (PL-MePS3) membranes (2500 RPM and 3000 RPM for 50 µm and 10 µm membranes, respectively, versus 6500 RPM for 2 µm thin membranes). The stiffness of PDMS membranes also increases with its diameter, as is also observed in other works [44].

Regarding *σ*_0_, 2 µm thin membranes have a value one order of magnitude higher than 10 and 50 µm thick membranes that show more similar values. Chambers of 5 mm diameter have *σ*_0_ values one order of magnitude smaller than 8 and 10 mm chambers. So thicker membranes of small diameters dissipate residual stress better than thinner ones, as expected for elastic materials.

Concerning the sensitivity of the chamber *Σ*, according to the data reported in Table 2, small diameter chambers (5 mm) are definitely more sensitive to pressure changes as the function of the loaded volume (first column data). Furthermore, compared to 8 and 10 mm chambers, they also show a lower elastic resistance to the deformation (lowest *E* values compared to larger chambers) and a lower residual stress *σ*_0_, which means higher deformability and less impact on the linearity and load capacity of the sensors.

However, since the deformation of chips of the same radius in response to an applied internal pressure represent a delicate balance between *d*, *E* and *σ*_0_ (see Equation (1)), it is important to calculate the parameter sensitivity of the membrane, *S*, to evaluate the more convenient device between the three 5 mm chamber devices with different membrane thicknesses: PL-MePS 1, 4 or 7. *S* is maximized when at small pressure changes Δ*P* corresponds to large-membrane deflection Δ*w*. Figure 4 is a plot of the deflection dependence *w* = *f*(*P*) for the 5 mm chambers with a higher sensitivity *Σ*. As reported in Figure 4, the highest membrane-sensitivity value *S* value is observed for the PL-MePS1 chip types and corresponds to (5.9 ± 0.41) µm/Pa. Hence, although characterized by a higher stiffness and residual stress, the chip more sensitive to small pressure changes was found to be the PL-MePS1. Indeed, considering the dependence of *P* from *w* of Equation (1), it seems that the reduction in the geometric parameter *d* impacts more than the increases in *σ*_0_ and *E* (that represent the mechanical properties of the membrane) on the deflection *w*, which increases for thinner membranes.

Given the performances of the different devices, it was decided to use PL-MePS1 as the sensor for the catalase study.

### 2.4. Catalase Studies

The study on catalase was performed at the loading volumes of 40 µL and 100 µL to detect a different range of catalase concentrations. These volumes were chosen since at V ≥ 40 µL, the deflection *w* of different sets of chips was found to be less dependent on the fluctuations due to the fabrication process, as discussed above and in Supplementary Materials (par. “Curve reproducibility”). In total, 20 µL or 50 µL of catalase at concentrations ranging from 50 to 500 nM and 0.5 to 100 nM were mixed with 20 µL or 50 µL of 30% H_2_O_2_ and inserted into the chamber of the MePS. The MePS was sealed as reported in the experimental section and the initial membrane deflection *w*_0_, due to the weight of the liquid, was calculated.

**Figure 5:**
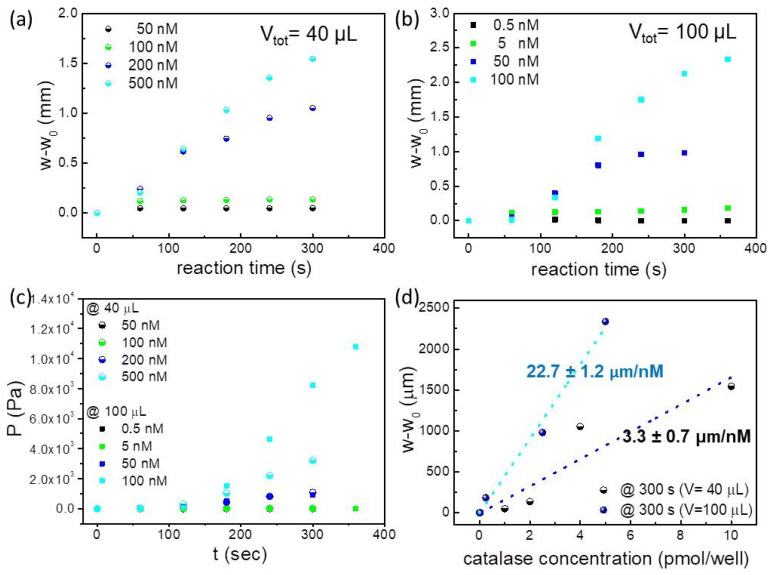
Deflection of the thin membrane during the catalase reaction as a function of time, for PL-MePS1 filled with (**a**) 20 µL of catalase solution (concentrations of 50, 100, 200 and 500 nM) and 20 µL of 30% H_2_O_2_ and (**b**) 50 µL of catalase solution (concentrations of 0.5, 5, 50 and 100 nM) and 50 µL of 30% H_2_O_2_. (**c**) Pressure evolution due to the catalytic reaction is reported as a function of time. (**d**) Deflection due to the reaction, detected at 300 s from the starting point of the reaction, is reported as a function of the solution molarity. The plot is used as a calibration curve for the sensitivity calculation. A sensitivity of 22.7 μm/nM is determined for the completely loaded chamber.

Figure 5a,b represent the evolution over the time of the value w-w_0_ which corresponds to the membrane deflection resulting from the generation of O_2_ during the reaction, while Figure 5c reports the pressure exerted during the reaction. As expected, the production of O_2_ steadily increases with rising catalase concentrations until it reaches a plateau. After 300 s for both loading volumes, the O_2_ amount remained nearly constant across all tested concentrations. The sensitivity, the linearity and the limit of detection (LoD) of the MePS sensor are calculated from the experimental calibration curve of deflection versus the catalase molarity (see Figure 5d) [49,50]. The sensitivity of the MePS, obtained at the equilibrium state (around 300 s from the reaction starting point), as the slope of the graph of Figure 5d, is (22.7 ± 1.2) µm/nM for a 100 µL loading volume and (3.3 ± 0.7) µm/nM for a 40 µL loading volume.

Differences in the PL-MePS1 sensitivity of catalase at different loading volumes can be attributed to the presence of a dead volume in the chamber not occupied by the liquid, and hence to an equilibrium between the O_2_ partitioned in the gas phase and O_2_ dissolved in the liquid phase, which may impact on the overall pressure generated inside the chamber and evaluated as membrane deflection.

**Figure 6:**
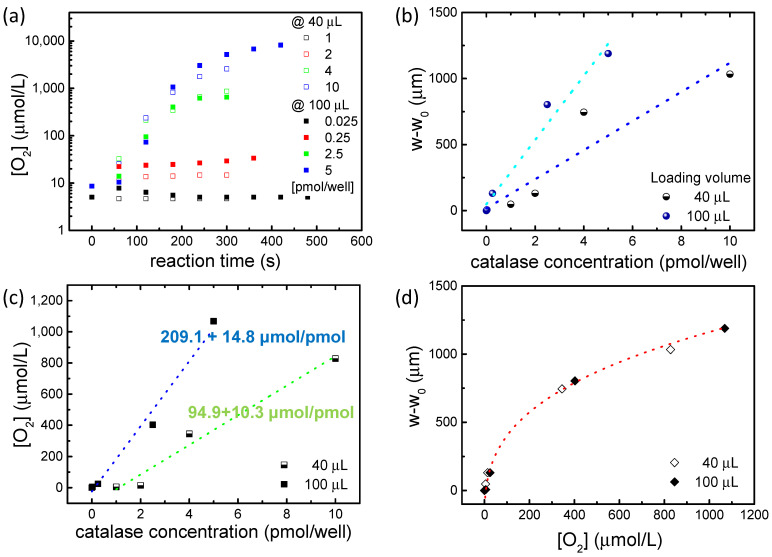
(**a**) Molar concentration of O_2_ ([O_2_], μmol/L) produced during the catalytic reaction at different catalase concentrations and for MePS microchambers filled with sample volumes of 40 μL and 100 μL. The O_2_ molar concentrations are obtained by measuring the deformation of the membrane during the reaction inside the MePS. (**b**) Deflections due the catalytic reaction and (**c**) O_2_ molar concentration at different catalase concentrations are analyzed at the reaction point of 180 s, corresponding to a linearity regime for the MePS sensor. A fitting over both datasets shows a twice-sensitivity of the sensor when the chamber is full at 100 μL. (**d**) The O_2_ molar concentration is a function of membrane deflection at 180 s from the start of the catalytic reaction for the two different load volumes.

Then, the LoD is obtained by dividing the sensor resolution with its sensitivity, and a value of 396 pM for the catalase PL-MePS1 when completely loaded is estimated. This LoD value generates a confidence interval of the analyte concentration for which sensor responses are reliable. The sensor independent linearity is of about ±5%, with a linear range from 396 pM up to at least 100 nM. The O_2_ moles produced during the catalytic reaction are calculated using the previously elaborated model for MePS sensors [18]. The O_2_ amount for each well is then analyzed as a function of membrane deflection at 180 s from the start of the catalytic reaction (corresponding to a timescale of the sensor linearity range). The graph in Figure 6a demonstrates that regardless of the sensor loading volume, MePS deflection can reveal the amount of oxygen produced by the reaction.

Sensors filled with a different volume of sample appear to have different behaviors when compared as a function of catalase molar concentration within the chamber (Figure 6b,c). The deflection produced during the reaction (Figure 6b) and the amount of O_2_ (Figure 6c), released in the linearity range of the sensor (at 180 s from the start of the reaction), are clearly dependent on the volume loaded into the MePS chamber, as shown by the fitting values in Figure 6b,c, and are correlated to the MePS sensitivity.

However, if the O_2_ concentration is plotted as a function of the reaction deflection for the two different loading volumes (as in Figure 6d), it is found that the normalized behaviors of the two sensors are analogous and comparable. A similar quantity of oxygen produced during the reaction corresponds to an analogous deformation of the membrane. More importantly, the trend of O_2_ produced follows a cubic-power law, as in the standard bulge test, with a fitting of R^2^ > 99%.

Therefore, regardless of how the sensor is filled, its linearity and correspondence with the expected behavior are confirmed. The MePS is reliable and its behavior is highly predictable and always respected, even when the chamber is partially or totally full. This result suggests the extreme design flexibility of the MePS according to the specific use. In addition to the possibility of modulating the geometric and membrane characteristics (thickness, diameter and Young’s modulus), it is also possible to modulate the load volume based on the concentrations to be detected. We have seen that, for example, for catalase concentrations higher than 1 pmol/well, even partially filling the chamber is sufficient. This allows us not to saturate the deflection of the membrane with the gravitational effect due to the weight of the liquid.

The transduction mechanism of MePS chips, based on direct pressure quantification, allows catalase to be estimated in a wider range than commercial colorimetric or enzyme-linked immunosorbent assays (from a few up to 100 ng/mL). The detectable range of catalase with our detection sensitivity varies from 396 pM up to at least 100 nM. A direct comparison of MePS sensitivity ((22.7 ± 1.2) µm/nM) and LoD (396 pM) with other sensors reported in the literature is rather difficult, considering that these values can vary significatively, depending on the catalase type and specific activity. However, although we used a catalase with a low specific activity (2000–5000 U/mg), we believe that our MePS-based chips show comparable results to many optical, amperometric and colorimetric sensors, but are less efficient compared to optical sensors based on liquid-crystal imaging (LoD in the fM or mU/mL range) [22,26,27,30,37,38,39,40]. Furthermore, considering that MePS technology can easily evolve towards low-cost portable devices (e.g., by smartphone integration), Point-of-Care tests for catalase detection in biofluids or immunoassays based on H_2_O_2_-fed biosensors can be implemented.

## 3. Materials and Methods

Soda–lime microscopic glass slides were provided by Pearl (Hong Kong, China); CLEVIOS PH 500 were purchased from Heraeus Clevios GmbH (Leverkusen, Germany) and toluene from J. T. Baker (Phillipsburg, NJ, USA). Sylgard-184; a two parts poly(dimethylsiloxane) (PDMS) elastomer, was purchased from Dow Corning (Midland, MI, USA); sulfuric acid (H_2_SO_4_, 98%), hydrogen peroxide (H_2_O_2_, 30%), catalase from bovine liver (2000–5000 units/mg protein) and potassium phosphate buffer (PB) were purchased from Sigma-Aldrich (Milan, Italy). Milli-Q water with a resistivity of 18.2 MΩ cm was used. PES syringe filters (0.45 μm) were purchased from Sartorius Stedim (Göttingen, Germany).

### 3.1. Fabrication of MePS Components

All the MePS chips reported in Table 1 consist of (1) reaction chambers of variable volumes and diameters and of (2) PDMS membranes of different thicknesses. To fabricate the chambers, a PDMS pre-polymer/curing agent ratio fixed to 10:1 in weight was mixed and poured in a glass Petri dish to obtain (6.5 ± 0.5) mm thick slides after curing at 140 °C for 15 min in an oven. After removal from the glass dish, reaction chambers of different diameters were produced using suitable punchers. PDMS-membrane preparation consists of several steps: (a) the cleaning of the glass slides using piranha solution (3:1 H_2_SO_4_:H_2_O_2_), then washing with milli-Q water and drying under nitrogen flow, (b) deposition by the spin coating of CLEVIOS PH 500 solution (filtered through the PES syringe filter) on the clean glass, acting as sacrificial layer, followed by baking on a hot plate at 120 °C for 5 min, (c) the preparation of a pre-polymer/curing agent solution (10:1 weight ratio) diluted with toluene (67% in weight) and (d) the spin-coating of PDMS-toluene solution on the sacrificial layer and curing in oven at 70 °C overnight.

### 3.2. MePS Assembly

MePS chips were assembled through two procedures: by plasma treatment (PL-MePS) and without plasma treatment (noPL-MePS). In PL-MePS chips, the punched slide and the PDMS thin membranes deposited on the glass substrate were both plasma-treated and put in conformal contact. Oxygen plasma was run in a low-pressure plasma system (Pico from Diener electronics, Ebhausen, Germany) by setting the parameters to the following: power at 100 W, oxygen flow at 240 sccm and pressure at 4 mbar for 60 s. After sealing with further pre-polymer/curing agent and polymerization at 140 °C for 15 min (using additional PDMS as glue), the slide-membrane assembly was put into water while stirring, to transfer the membrane on the chambers and remove the glass substrate. Then, washing by pure water was performed to remove CLEVIOS PH 500 residues on the membrane side that was in contact with the glass substrate. In noPL-MePS chips, the plasma treatment was replaced with the “mortar layer” method [51] using a 10 µm layer of PDMS diluted in toluene deposited by spin-coating on a cleaned glass substrate. The punched slide and the edges of the PDMS membranes were put in contact for 10 s with the PDMS uncured layer and bonded at 140 °C for 15 min in an oven. No additional sealing with PDMS as a glue was used prior to the membrane transfer step.

### 3.3. Bulge Test

To test the sensitivity of the MePS chips reported in Table 1, water was dropped into the chamber with steps of 10 or 20 μL that locally deformed the membrane. As the volume of water increased, the membrane underlying the droplet extended under the gravitational force. The membrane deflection (w) of the MePS chips was monitored using a CAM 200 (KSV Instruments Ltd., Espoo, Finland) instrument, measured by using ImageJ 1.46r software analysis and plotted versus water volume (see Figure 1a). The error on the w estimation was ±3 μm.

### 3.4. Catalase Experiments

For the experiments, the complete sensor was directly loaded with the test solutions. The solutions of catalase in 50 mM PB used were 0.5, 5, 50, 100, 200 and 500 nM. In total, 50 µL or 20 µL of catalase were mixed in the reaction chamber of MePS chips, respectively, with 50 µL or 20 µL of 30% H_2_O_2_; then, the chips were immediately sealed, and the membrane deflection was imaged using the camera and followed at the timepoints of 60 s up to 5–8 min (see Figure S5).

## 4. Conclusions

Membrane-based sensors are highly sensitive transducers for detecting pressure changes within confined microsystems. Reactions generating gases and triggering pressure shifts can serve as catalyst detectors and as amplification mechanisms for analyte detection in bioassays. One key reaction generating O_2_ involves the disproportionation of H_2_O_2_, a process naturally catalyzed by the enzyme catalase but also achievable through various synthetic catalase-like compounds.

In humans, catalase has been linked to numerous physiological and pathological conditions. Recent studies have unveiled unknown functions of this enzyme as well as the importance of direct catalase detection in biofluids for diagnostic purposes. Catalase-like systems have been also used in biological assays as amplification methods to detect analytes with a concentration proportional to the catalyst by estimating the volume of the gaseous products produced. In membrane-based sensors, these gaseous products induce a pressure increase, measured by assessing membrane deflection.

In this work, we report the fabrication of an effective PDMS membrane-based sensor (MePS) obtained from optimized parameters of Young’s modulus *E*, residual stress *σ*_0_ and the sensitivity of both chamber size *Σ* and membrane *S*. Several MePSs have been fabricated and characterized as sensors, modifying membrane structural parameters (such as membrane thickness or chamber diameter) and monitoring their role in sensing during a catalytic reaction.

Effects besides that of an oxygen plasma treatment on the reliability and response linearity of the membrane device used as a sensor have been clarified. Oxygen plasma treatment was found to be crucial for enabling a uniform deflection of the membrane and a reproducible response, mainly due to the resulting increased hydrophilicity of the membrane.

Finally, our results demonstrate that the sensitivity of the MePS is dependent on the loading volume, although its linearity and correspondence with the expected behavior are always preserved. By loading 100 µL of reactive solution, a sensitivity of (22.7 ± 1.2) µm/nM was obtained with a LoD of 396 pM and an independent linearity of ±5% in a wide range up to at least 100 nM. According to these results, the MePS technology appears very sensitive to volumetric changes and is amenable for portability, improvements in sensitivity and LoD (i.e., using more performant catalase-like systems) and integration into multifunctional chips because of its easy miniaturization.

## Figures and Tables

**Table 1:** Types of MePS chips produced with main features like chamber diameter (2r), membrane thickness (d) and chamber internal volume (V_in_).

MePS Chips	2r (mm)	d (µm)	Vin (µL)
PL-MePS1	5	2	120
noPL-MePS1	5	2	120
PL-MePS2	8	2	340
PL-MePS3	10	2	500
noPL-MePS3	10	2	500
PL-MePS4	5	10	120
PL-MePS5	8	10	340
noPL-MePS5	8	10	340
PL-MePS6	10	10	500
PL-MePS7	5	50	120
PL-MePS8	8	50	340
PL-MePS9	10	50	500

**Table 2:** Young’s modulus *E*, residual stress *σ*_0_ and sensitivity values of the chamber *Σ* for all the chips as extracted from the pressure-deflection data.

d (µm)	2r = 5 mm	2r = 8 mm	2r = 10 mm
2	PL-MePS1	PL-MePS2	PL-MePS3
	*E* = 1.75 ± 0.90 Mpa *σ*_0_ = 0.094 ± 0.003 MPa *Σ* = 0.525 ± 0.003 Pa/µL	*E* = 3.28 ± 0.46 MPa *σ*_0_ = 0.220 ± 0.055 MPa *Σ* = 0.2090 ± 0.0008 Pa/µL	*E* = 5.32 ± 0.92 MPa *σ*_0_ = 0.310 ± 0.094 MPa *Σ* = 0.1370 ± 0.0008 Pa/µL
10	PL-MePS4	PL-MePS5	PL-MePS6
	*E* = 1.51 ± 0.98 Mpa *σ*_0_ = 0.004 ± 0.002 MPa *S* = 0.525 ± 0.002 Pa/µL	*E* = 1.73 ± 0.67 Mpa *σ*_0_ = 0.051 ± 0.007 MPa *S* = 0.2070 ± 0.0003 Pa/µL	*E* = 2.11 ± 0.74 Mpa *σ*_0_ = 0.068 ± 0.008 MPa *S* = 0.1360 ± 0.0004 Pa/µL
50	PL-MePS7	PL-MePS8	PL-MePS9
	*E* = 1.23 ± 0.78 Mpa *σ*_0_ = 0.003 ± 0.001 MPa *S* = 0.513 ± 0.002 Pa/µL	*E* = 1.43 ± 0.81 MPa *σ*_0_ = 0.074 ± 0.003 MPa *S* = 0.2060 ± 0.0008 Pa/µL	*E* = 1.59 ± 0.51 MPa *σ*_0_ = 0.011 ± 0.002 MPa *S* = 0.1340 ± 0.0002 Pa/µL

## Data Availability

All raw data and experimental details are available upon request.

## Supplementary Materials

The following supporting information can be downloaded at: https://www.mdpi.com/article/10.3390/molecules29071506/s1, Figure S1: Plots from curves of Figure 3b to calculate (a) the residual stress *σ*_0_ and Young’s modulus *E*, (b) Sensitivity of chamber dimension *Σ* for PL-MePS4-6 with membrane thickness of 10 µm and diameter of 5, 8 and 10 mm respectively; Figure S2: Plots from curves of Figure 3c to calculate (a) the residual stress *σ*_0_ and Young’s modulus *E*, (b) Sensitivity of chamber dimension *Σ* for PL-MePS7-9 with membrane thickness of 50 µm and diameter of 5, 8 and 10 mm respectively; Figure S3: Images of membrane deflection of MePS1 (diameter of the chamber 8 mm and membrane thickness 2 µm) (a) treated with oxygen plasma (PL-MePS1) with addition of 80 µL of water and (b) without oxygen (noPL-MePS1) plasma treatment and with addition of 20 µL of water. Images of membrane deflection of PL-MePS3 (diameter of the chamber 10 mm and membrane thickness 2 µm) (c) treated with oxygen plasma (PL-MePS3) with addition of 200 µL of water and (d) without oxygen (noPL-MePS3) plasma treatment and with addition of 10 µL of water; Figure S4: (a) Bulge tests performed using three different PL-MePS1 (chamber diameter 5 mm; membrane thickness 2 µm) and their corresponding data analyses to evaluate (b,c) the Young’s Modulus *E* from the *P* versus *w* curves; Figure S5: Pictures of (a) the holder in plexiglass in which MePSs with three separate chambers can be included to be well sealed. (b) Set up to measure membrane deflection consisting in a high resolution camera and a software for acquiring and elaborating the pictures showing the membrane deflection.
