# Supplementary material for: Catalase Detection via Membrane-Based Pressure Sensors
Source: Molecules. 2024 Mar 28;29(7):1506. doi: 10.3390/molecules29071506 (PMC11013101; doi:10.3390/molecules29071506)
Supplement: Supplementary file 1 [file molecules-29-01506-s001.zip › molecules-2890269-supplementary.pdf]

Supplementary Information

Catalase Detection via Membrane-Based Pressure Sensors

Monica Bianco [1], Alessandra Zizzari [1], Elisabetta Perrone [1], Diego Mangiullo [1], Marco Mazzeo [2], Ilenia Viola [3,*] and Valentina Arima [1,*]

[1] CNR NANOTEC—Institute of Nanotechnology, c/o Campus Ecotekne, Via Monteroni, 73100 Lecce, Italy; monica.bianco@nanotec.cnr.it (M.B.); alessandra.zizzari@nanotec.cnr.it (A.Z.); elisabetta.perrone@nanotec.cnr.it (E.P.); diego.mangiullo@nanotec.cnr.it (D.M.)
[2] Department of Mathematics and Physics "E. De Giorgi", University of Salento, 73100 Lecce, Italy; marco.mazzeo@unisalento.it
[3] CNR NANOTEC—Institute of Nanotechnology, S.Li.M Lab, c/o Department of Physics, Sapienza University, P.le A. Moro 5, 00185 Rome, Italy
* Correspondence: ilenia.viola@nanotec.cnr.it (I.V.); valentina.arima@nanotec.cnr.it (V.A.)

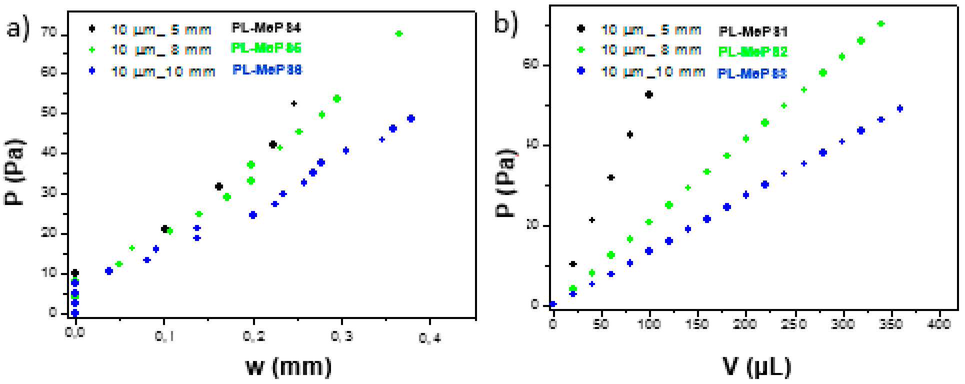

Figure S1. Plots from curves of Figure 3b to calculate a) the residual stress σ_0 and Young's modulus E, b) Sensitivity of chamber dimension Σ for PL-MePS4-6 with membrane thickness of 10 μm and diameter of 5, 8 and 10mm respectively.

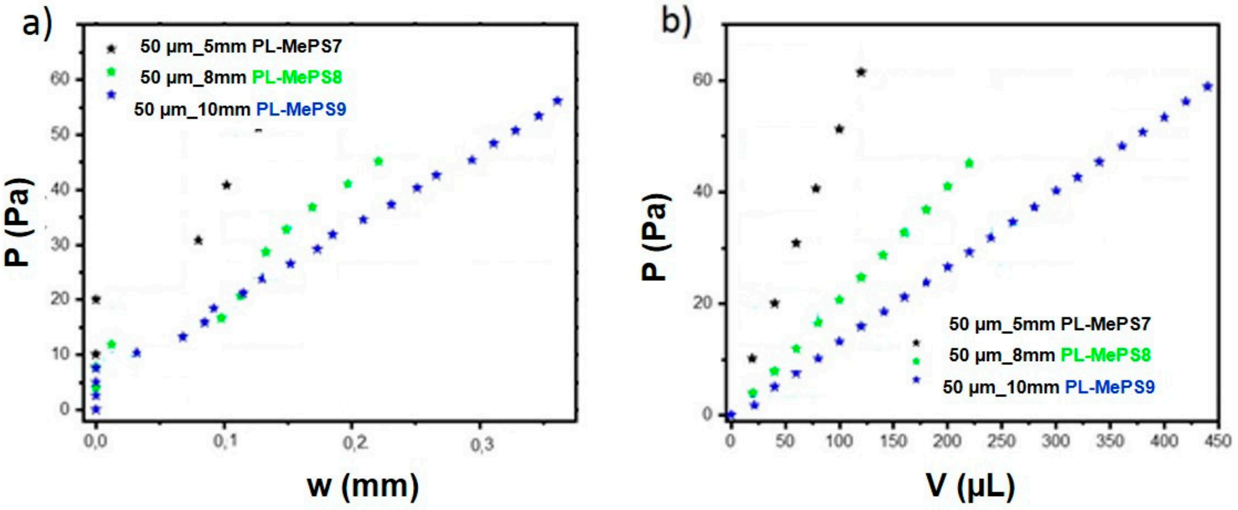

Figure S2. Plots from curves of Figure 3c to calculate a) the residual stress σ_0 and Young's modulus E, b) Sensitivity of chamber dimension Σ for PL-MePS7-9 with membrane thickness of 50 µm and diameter of 5, 8 and 10 mm respectively.

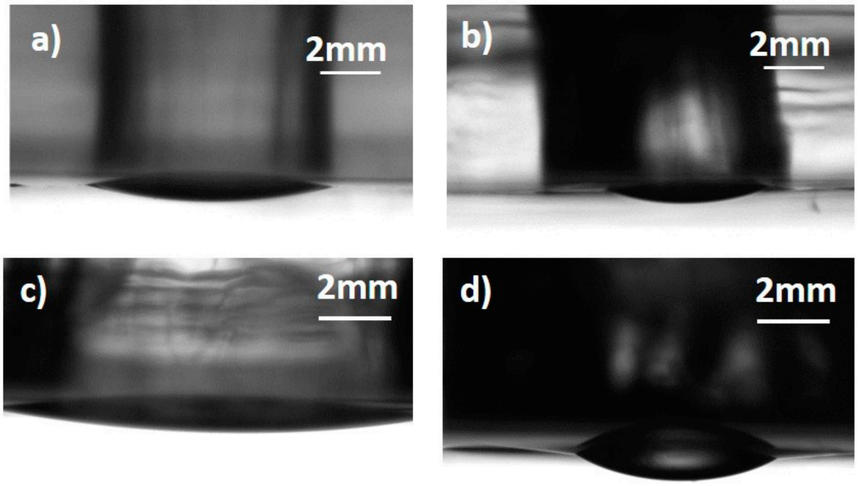

Figure S3. Images of membrane deflection of MePS1 (diameter of the chamber 8 mm and membrane thickness 2 µm) a) treated with oxygen plasma (PL-MePS1) with addition of 80 µL of water and b) without oxygen (noPL-MePS1) plasma treatment and with addition of 20 µL of water. Images of membrane deflection of PL-MePS3 (diameter of the chamber 10 mm and membrane thickness 2 µm) c) treated with oxygen plasma (PL-MePS3) with addition of 200 µL of water and d) without oxygen (noPL-MePS3) plasma treatment and with addition of 10 µL of water.

Plasma-induced effects on membranes. Figure 1a shows the bulge tests acquired on two MePS5 devices assembled with different fabrication methods (PL- and noPL-). Figures 1b and its insets show pictures respectively of the PL- and noPL-MePSs loaded with the same volume of water. The deformation of the two devices is very different; PL- one is very homogenous, with a minimum deflection easily detectable and quantifiable. NoPL-MePS shows an uneven deformation with the water amount concentrated in the center of the membrane. In this case, the estimate of the maximum deflection w, although possible, is not correct since the non-hydrophilic membrane does not distribute the water homogeneously over the entire surface of the membrane. The addition of larger volume of water lead to a more uniform deformation of the membrane but with negligible influences on the observed maximum deflection value. This is clearly visible in the plot of Figure 1b. The bulge test of PL-MePS shows a linear increase at added water volumes (curve with full black dots), while the curve of the noPL-MePS shows a very low increment of deflection at water amount addition

(empty dots curve). The dashed lines, as a guide for the eye in Figure 1b, show a linear response to inputs only for PL-MePS.

This difference between PL and noPL-MePSs was confirmed for all the chips (see also optical images in Figure S4 for PL-MePS1 and PL-MePS3) but is less evident for the 5 mm diameter reaction chambers due to the lower surface area at the interface with water. Therefore, considering the lower sensitivity of noPL membranes to water addition and nonlinear behavior, the curve fittings and further studies have been performed only using PL-MePSs.

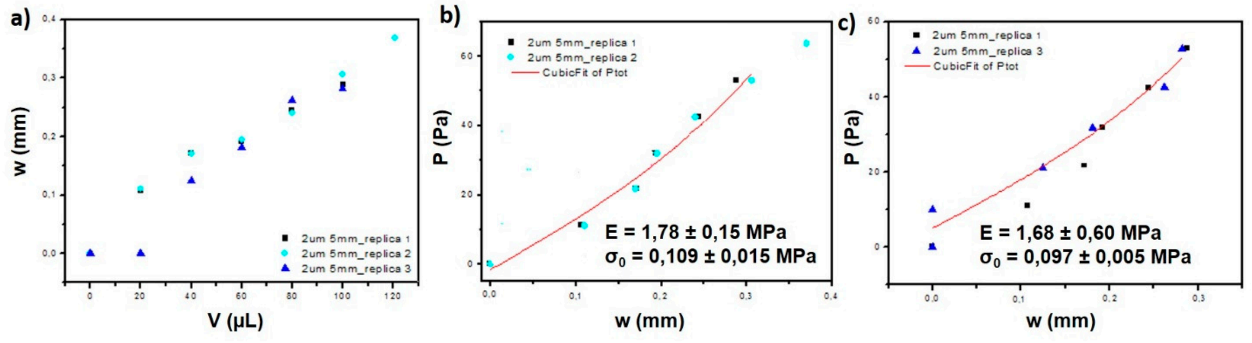

Figure S4. a) Bulge tests performed using three different PL-MePS1 (chamber diameter 5 mm; membrane thickness 2 µm) and their corresponding data analyses to evaluate b) c) the Young's Modulus E from the P versus w curves.

Curve reproducibility. As preliminary experiments, the reproducibility of the acquired curves was tested by performing the same experiments using three different chips of PL-MePS1 type (see Figure S5a and S5b). The bulge tests appear quite reproducible at water volumes ≥ 40 µL which corresponds to loading pressures ≈ 30 Pa. At lower pressures, some differences due to the fabrication process cause a higher inertia to mechanical deformation [1]. Indeed, in previous works, a low reproducibility of the elastic behavior of thin PDMS membranes was attributed to the presence of ridges or to a not truly flat surface. Ridges are supposed to form during the peeling off of the membrane from the substrate and to become permanent after the oxygen plasma exposure; on the other hand, a no-uniform coating on the substrate or dirt particles during the preparation of the solutions may generate a not fully flat membrane [1]. Here, although we do not peel off the membranes from the substrate, since we gently remove them by dissolution of the sacrificial layer, some ridges are formed and also we cannot exclude that dirt particles or aggregates deposit on the sacrificial layer, thus reducing the membrane flatness and uniformity. A proper calibration of each MePSs before use may eliminate this problem.

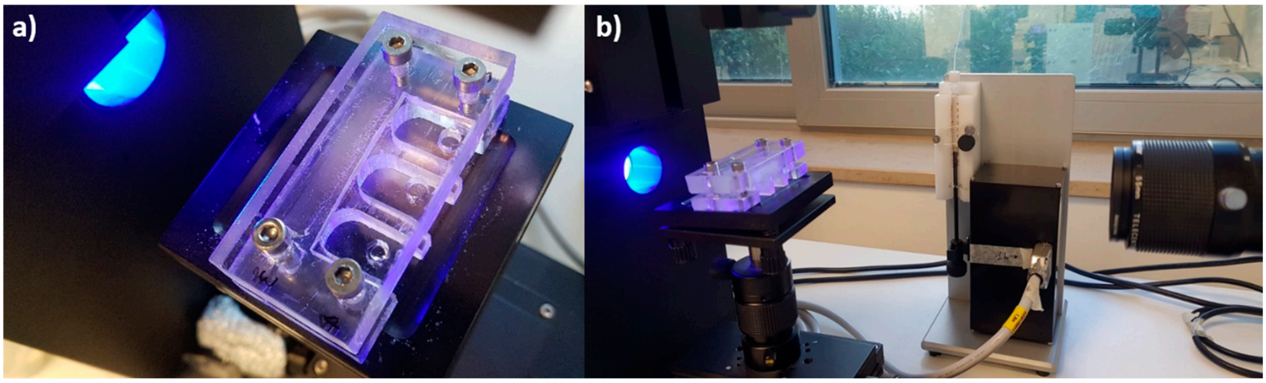

Figure S5. Pictures of a) the holder in plexiglass in which MePSs with three separate chambers can be included to be well sealed. b) Set up to measure membrane deflection consisting in a high resolution camera and a software for acquiring and elaborating the pictures showing the membrane deflection.

Reference

1. A.L. Thangawng, R.S. Ruoff, M.A. Swartz, M.R. Glucksberg, An ultra-thin PDMS membrane as a bio/micro-nano interface: Fabrication and characterization, Biomed Microdevices. 9 (2007) 587–595. https://doi.org/10.1007/s10544-007-9070-6.
